# Recent Advances and Potential Future Applications of MALDI-TOF Mass Spectrometry for Identification of Helminths

**DOI:** 10.3390/diagnostics12123035

**Published:** 2022-12-03

**Authors:** Issa Sy, Lucie Conrad, Sören L. Becker

**Affiliations:** 1 Institute of Medical Microbiology and Hygiene, Saarland University, 66421 Homburg, Germany; 2 Swiss Tropical and Public Health Institute, 4123 Allschwil, Switzerland; 3 University of Basel, 4001 Basel, Switzerland

**Keywords:** diagnosis, helminths, cestodes, nematodes, trematodes, matrix-assisted laser desorption/ionization time-of-flight (MALDI-TOF), mass spectrometry (MS)

## Abstract

Helminth infections caused by nematodes, trematodes, and cestodes are major neglected tropical diseases and of great medical and veterinary relevance. At present, diagnosis of helminthic diseases is mainly based on microscopic observation of different parasite stages, but microscopy is associated with limited diagnostic accuracy. Against this background, recent studies described matrix-assisted laser desorption/ionization time-of-flight (MALDI-TOF) mass spectrometry as a potential, innovative tool for helminth identification and differentiation. MALDI-TOF mass spectrometry is based on the analysis of spectra profiles generated from protein extracts of a given pathogen. It requires an available spectra database containing reference spectra, also called main spectra profiles (MSPs), which are generated from well characterized specimens. At present, however, there are no commercially available databases for helminth identification using this approach. In this narrative review, we summarize recent developments and published studies between January 2019 and September 2022 that report on the use of MALDI-TOF mass spectrometry for helminths. Current challenges and future research needs are identified and briefly discussed.

## 1. Introduction

Helminths are parasitic worms that give rise to considerable disease burden in humans and animals. From a medical perspective, the major helminths are classified into three distinct groups, based on their external shape and the host organ they are located in: cestodes (tapeworms), nematodes (roundworms), and trematodes (flukes) [1]. They represent some of the most common infectious agents of humans, and helminthiasis is a major neglected tropical disease (NTD), which affects in particular individuals in the Global South [2,3]. A precise diagnosis of these infectious agents is crucial for control efforts and individual patient management. Currently used parasitological methods frequently rely on light microscopy and have limited diagnostic accuracy, especially in light-intensity infections. Moreover, some species such as hookworms [4] or *Taenia* spp. [5] have almost identical eggs and are thus indistinguishable on microscopic identification. While new methods have been proposed [6,7], different challenges remain. Molecular methods such as polymerase chain reaction (PCR)-based approaches can also be employed for helminth diagnosis [8]. These methods have a high sensitivity but are still expensive, require specific technical expertise, and are not really adapted for routine application, especially in resource-limited or developing countries, where helminths are endemic. Additionally, only a very limited set of PCR assays targeting helminths are commercially available thus far. In many parts of the world, where helminth infections are nowadays less common, expertise in correct morphological identification is also waning. Hence, there is a need for innovative, examiner-independent techniques for helminth identification.

For more than one decade, matrix-assisted laser desorption/ionization time-of-flight (MALDI-TOF) mass spectrometry (MS) has become the widely accepted gold standard method in many clinical microbiology laboratories, and it is routinely used for the identification of bacteria [9,10] mycobacteria [11,12] and fungi [13]. This technology is based on the rapid and accurate identification of microorganisms using an analysis of pathogen-specific protein spectra profiles, which are measured and identified through comparison to an existing database [14]. Of note, MALDI-TOF MS analyses are not only limited to protein profile studies. Recent studies emphasized the possible use of MALDI-TOF MS for lipidological analysis and for prediction of antibiotic resistance pattern [15,16,17,18]. The principle of MALDI-TOF MS can be briefly described as follows: a sufficient amount of bacterial/fungal biomass (or their protein extracts; e.g., taken directly from an agar plate) is placed onto the MALDI plate and overlaid with a matrix (e.g., HCCA: alpha-cyano-4-hydroxycinnamic acid). The mixture (sample + matrix) is crystalized and then subjected to a laser beam. The heat energy of the laser ionizes the sample, and its molecules migrate through an electric vacuum field in the MALDI-TOF mass spectrometer. The ions are separated during the migration according to their mass-to-charge ratio (time-of-flight). Once they reach a detector, a specific spectra profile is generated and compared to a commercially available reference spectra database [19]. Within seconds, a result is generated as so-called log score value (LSV), which estimates the accuracy of identification. For bacteria, any LSV ≥ 1.70 is considered as genus-specific identification and LSVs ≥ 2.00 are considered as species-specific identification.

**Figure 1:**
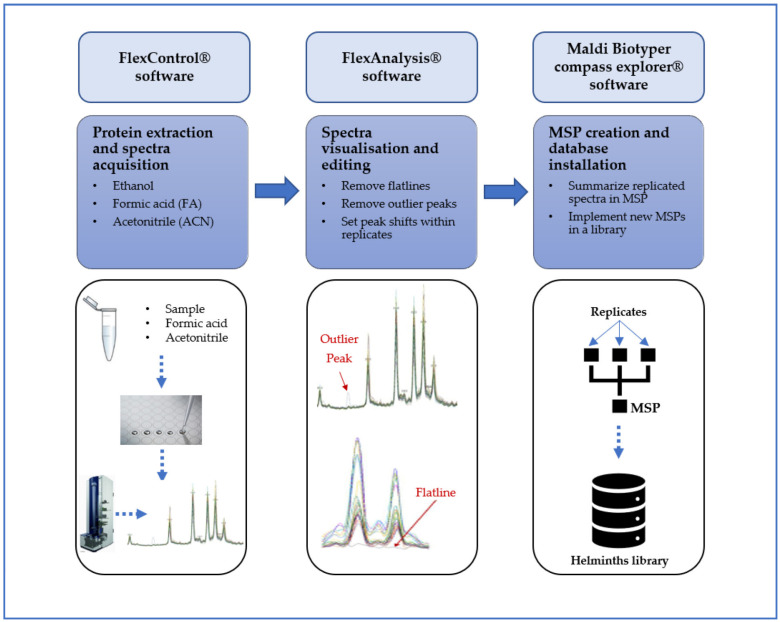
MSP creation and database installation using MBT compass explorer software version 3 (Bruker Daltonics, Bremen, Germany).

In contrast to bacteria and fungi, there is currently neither a commercial library for an MALDI TOF-based identification of parasites nor a standardized protocol for measurements of parasitic elements. However, the idea of employing MALDI-TOF MS for parasites and especially helminths is gaining more and more interest, and different research groups have worked on this topic [20]. As a first prerequisite, it is important to develop a spectral database for any subsequent identification. The MALDI-TOF MS apparatus released by the German company Bruker (Bruker Daltonics, Bremen, Germany) allows to create reference spectra (also called MSPs: main spectra profiles) using the Maldi Biotyper (MBT) compass explorer software (MSP creation protocol V1.1; Bruker Daltonics; Bremen, Germany). Figure 1 indicates a summary of the different steps that are required for MSP creation and database development and installation. Once the helminth-specific database is installed, new samples can be identified very rapidly using a simple procedure. Therefore, due to its accuracy, ease of use, and cost-effectiveness of reagents, the application of MALD-TOF MS for helminths identification offers a true advantage in comparison to microscopy or PCR-based methods.

In this article, we review the recent advances and highlight potential future applications of MALDI-TOF MS for the identification of helminths. Given the increasing availability of mass spectrometers in reference laboratories of low- and middle-income countries as well as the waning knowledge regarding identification of helminths among microbiologists in high-income settings, detection of helminths by MALDI-TOF MS might be advantageous in a host of different settings.

## 2. Materials and Methods

A literature review was conducted to assess the current state of MALDI-TOF MS as a diagnostic tool for helminths. As a previous review had included manuscripts published on this topic until late 2018 [20], we searched published articles from January 2019 to September 2022 in the following databases: PubMed/MEDLINE and Google Scholar. The search strategy included combinations of the following keywords: “matrix-assisted laser desorption/ionization time-of-flight (MALDI-TOF) mass spectrometry (MS)”, “helminth”, “nematode”, “cestode” and “trematode”. The literature research was independently carried out by all three authors, and subsequently synthesized by the first two authors to generate this narrative review. No language restriction was used during the search.

## 3. Specific Application of MALDI-TOF MS for Different Helminths

### 3.1. Cestodes

We found only two studies that reported on the use of MALDI-TOF MS for cestodes. One study focused on the analysis of specific proteins from larval stages, while the second report investigated the identification of direct identification of adult worms (proglottids).

Diaz-Zaragoza et al. [21] examined the proteomic profiles of larval stages of the tapeworm *Taenia crassiceps* in a murine intraperitoneal model to assess the immunological T helper cell (Th) response during cysticercosis, in particular the differential Th1 and Th2 responses. After two-dimensional gel electrophoresis (2DE), proteins were selected and identified by MALDI-TOF MS. It was shown that *T. crassiceps* cysticerci are able to modify their proteome depending on the activation of Th1 or Th2 response in the course of infection. Furthermore, the study assessed how cysticerci interact with their environment as well as which mechanisms allow for infection persistence in the host.

A more recent study carried out by Wendel and colleagues [22] showed that parts of adult cestodes (proglottids) can also be identified by MALDI-TOF MS. In this study, the authors showed that the beef tapeworm *Taenia saginata* can be reliably diagnosed by MALDI-TOF MS. For this purpose, *T. saginata* proglottides isolated from stool samples of infected humans were subjected to protein extraction and MALDI-TOF MS analysis. Protein spectra were generated under repeatability and reproducibility conditions and were analyzed in order to create species-specific MSPs to obtain an in-house database. Subsequently, samples were measured and analyzed by this database, leading to reliable LSVs in 97.2% to 99.7% of the analyzed spectra. Hence, this study showed the potential utility of such a diagnostic approach in humans but was limited by the low number of samples and the lack of external validation using new independent samples.

### 3.2. Nematodes

The utilization of MALDI-TOF MS for nematode identification has been reported by recent studies of either pathogen-associated proteins or the pathogen itself at different developmental stages (adults, larvae, or eggs) [23,24]. Bredtmann and colleagues [25] showed the possible differentiation of adult worms of two closely related cyathostomin species, i.e., *Cylicostephanus longibursatus* and *Cylicostephanus minutus*. In this study, the authors collected adult samples from horses (intestines, cecum, and feces) and performed MALDI-TOF analysis using an in-house MSP library. High-quality and repeatable spectra were acquired with fresh samples collected during necropsy, while samples recovered from feces after anthelminthic treatment gave spectra with low quality. The database validation test showed correct species identification of 89% (144/162) of all measured spectra. Similarly, Nagorny and colleagues [26] reported a MALDI-TOF MS-based identification of five adult female *Dirofilaria repens* originating from an infected human, and five *Dirofilaria immitis* isolated from dogs. They showed a concordance rate of 100% and 70% for *D. repens* and *D. immitis*, respectively. More recently, another study carried out by Rivero and colleagues [27] emphasized the identification of *Trichuris* spp. using MALDI-TOF MS. In this study, the authors analyzed different parts (esophagus and intestine) of five whipworms to create reference MSPs. Subsequently, they queried 20 new whipworms (18 esophagus and 20 intestines) to the reference database for external validation, resulting in nine esophagus samples (50%) and 16 intestines samples (80%) as being correctly identified (*Trichuris suis* with LSVs ≥ 2.00). However, a dendrogram analysis revealed that a differentiation between both parts (esophagus and intestine) was not possible by means of MALDI-TOF MS.

In addition to the identification of adult worms, other studies have also investigated the application of MALDI-TOF MS for the identification of larval stages. Karadjian and colleagues [28] analyzed larvae of *Trichinella* spp. that were isolated from different hosts (i.e., pigs, wild boars, red foxes, raccoon dogs, and horses) and different geographical areas (e.g., Italy, France, Romania, Serbia, Hungary, Serbia, Poland, Germany, Norway, Finland, Bulgaria, Tasmania, Russia, Japan, China, USA, Argentina). They reported a correct identification rate with LSVs ≥ 2.00 in 100% (70/70), 96.1% (49/51), and 83.3% (5/6) for *T. spiralis*, *T. britovi* and *T. nativa*, respectively. The authors also reported no close relationship between the *T. nativa* strains isolated from Germany, Norway, and Russia. Based on this observation, they suggest a possible difference in protein spectra associated with the geographical location. Dendrogram analysis also revealed varying distance levels of *T. britovi* and *T. nativa*, which may indicate intraspecific differences in both species. Taken together, this study underscored the possibility of correctly identifying *Trichinella* spp. based on protein spectra profiles. However, the study was limited by the use of rather old reference strains, and there is a need to also include additional spectra from more recent strains into the MSP database. Likewise, Marzano and colleagues [29] described the use of MALDI-TOF MS profiling as a new diagnostic tool for *Anisakis* spp. larvae isolated from salmons. Clustering analysis performed on spectral data obtained from five *Anisakis* spp. larvae showed a phenotypic variability between the five larval samples (larvae 1 and 4 clustered together and formed a clade with larva 5; while larvae 2 and 3 formed a different cluster), possibly indicating the discriminatory power using protein fingerprints. In addition, the authors also identified 19 signals (peaks), which may be used as “fingerprinting classifier” specific to *Anisakis* spp. This study emphasized the potential use of this method, and this may lead in future to MALDI-TOF MS-based identification of *Anisakis* larvae obtained from e.g., the gastrointestinal tract of infected human patients.

Apart from using MALDI-TOF MS as a new tool for the identification of adult worms or larvae, other studies focused on the characterization and identification of specific nematode proteins. Khanmohammadi and colleagues [30] studied the characterization of immunogenic proteins of *D. immitis* using MALDI-TOF MS. Herein, authors collected adult worms from six infected dogs and cultivated them in cell culture flasks containing roswell park memorial institute (RPMI)-1640 medium and other substances. Afterwards, the medium was submitted to successive steps of centrifugation, filtration, homogenization, and sonication in order to retrieve the in vivo secretome (somatic and excretory/secretory (E/S) protein extracts). Immunoreactive protein bands detected by Western blots were then excised and analyzed by MALDI-TOF MS. The authors used UniProt and European molecular biology laboratory (EMBL) databases to characterize the identified proteins. Nine and eight proteins were identified in the somatic (polyprotein antigen, P22u, galectin, etc.) and E/S (pepsin inhibitor Dit33, superoxide dismutase, etc.) extracts, respectively, including six common proteins. Martini and colleagues [31] reported on the presence of N-glycans from *D. immitis* of up to 7000 Da using MALDI-TOF MS (both linear and reflector mode) in combination with other techniques (e.g., high-performance liquid chromatography (HPLC), enzymatic digestion, etc.). These N-glycans contain features that can be involved in immunomodulation or help to avoid the immune response. Similarly, Wang and colleagues [32] also studied the N-glycome and N-glycoproteome of the ruminant nematode *Haemonchus contortus*. They reported a total of 291 N-glycosylated proteins occurring primarily in the intestine and gonads. In the same direction, Petralia and colleagues [33] investigated the glycans expressed by the filarial nematode *Brugia malayi* at different stages of development (microfilariae, third-stage larvae (L3), and adult worms (males and females)) using MALDI-TOF MS combined with glycan sequencing, tandem mass spectrometry (MS/MS) and glycan microarrays. They identified several antigen motifs such as phosphorylcholine and terminal glucuronic acid. Antibody responses with a significant level of IgG were also detected during microarray screening.

### 3.3. Trematodes

In a French study by Huguenin and colleagues [34], MALDI-TOF MS was employed to identify cercarial stages of different trematodes. To this end, the authors collected different snails as they are the intermediate hosts of many trematodes. Cercariae from the genera Lymnaeidae and Planorbidae were released after specific hatching tests. Using molecular identification, the detected cercariae included the following species: *Trichobilharzia anseri*, *Diplostomum pseudospathaceum*, *Tylodelphys* sp., *Australapatemon* sp., *Cotylurus* sp., *Posthodiplostomum* sp., *Parastrigea* sp., *Alaria alata*, *Echinostoma revolutum*, *Petasiger phalacrocoracis*, *Echinoparyphium* sp. and *Plagiorchis* sp. MALDI-TOF MS analysis was performed for each species and the obtained spectra were processed. Based on this, reference spectra of all species except *Parastrigea* sp. and *Cotylurus* sp. were generated and integrated into an in-house database. Subsequently, the newly created database was evaluated by ‘blind tests’ with new samples. Of the analyzed spectra acquired from species in the database, 68.36% achieved an LSV of 1.7, and thus, reliable genus identification. The sensitivity was 81.7% and the specificity was 100%. After adding reference spectra of *Cotylurus* sp., 65.78% achieved an LSV of ≥1.7, and 100% were correctly identified. In the same way, Kästner et al. [35] conducted a study to identify *A. alata* mesocercariae isolated from meat by MALDI-TOF MS. When wild boar meat is examined for *Trichinella*, mesocercariae of *A. alata* are frequently found. For this study, mesocercariae have been isolated in several countries from muscle samples from different hosts, including wild boars, lynxes, and amphibians. After developing an applicable protocol for protein extraction, MALDI-TOF analysis was performed. Spectra were analyzed and spectra of high quality were selected. A total of 10 mesocercariae were used to integrate species-specific MSPs into the database. Unknown spectra were then compared to the new database for validation. The protocol was tested in two different laboratories and proved to be reproducible. Thirty-six of 38 mesocercariae had LSVs of above 2.0 in all three individual spectra, which demonstrated the repeatability of the chosen approach. In addition, spectra from *Opisthioglyphe ranae* larvae were also analyzed by MALDI-TOF MS and queried to the new database. The obtained score values (≤1.7) demonstrate the capacity to distinghuish between different trematodes species using their spectra profiles. The protein spectra of the mesocercariae in this study, however, did not correspond to those previously reported [34], which could be caused by the different larval stages as well as the host species or the slightly different protein extraction protocol. More recently, Huguenin and colleagues [36] also reported the identification of cercariae of schistosomes using MALDI-TOF. In the study, different *Schistosoma* species were included: *S. haematobium*, *S. mansoni*, *S. bovis*, *S. rodhaini*, and the hybrid *S. bovis* × *S. haematobium*. After spectra acquisition and analysis, MSPs were generated and added to the database. It was possible to distinguish between the individual species. Blind tests showed an accuracy of 94% and a specificity of 99.56%. The discrimination between *S. haematobium* and the hybrid species (*Corsican hybrids*), however, remained a challenge. Yet, the authors also used machine learning algorithms combined with MALDI-TOF MS spectra in order to increase the accuracy of the discrimination to 97%.

In addition to the use of cercariae, MALDI-TOF has also been employed for a rapid diagnosis and early screening of schistosomiasis in serum samples. Huang and colleagues [37] studied the identification of newly developed advanced schistosomiasis (NDAS), using 30 serum samples stemming from infected patients, as well as 30 sera from healthy people as control group. Protein extracts obtained from these two groups were subjected to MALDI-TOF MS after a weak cation exchange beads (MB-WCX) treatment, using the ClinProTool software. A comparison analysis revealed a total of 14 peaks (four upregulated peaks (increase of the intensity); and 11 downregulated peaks (decrease of the intensity)) as important for the discrimination of the two groups and were established as proteomic detection patterns (PDP). The authors also evaluated the specificity and sensitivity of the PDP by subjecting new mass fingerprints (spectra) of sera from 50 NDAS and 100 healthy controls for a blind test. They reported a sensitivity of 100% and a specificity of 92%. The PDP created in this study could potentially be used to identify NDAS patients, and the peaks might possibly serve as future biomarkers for diagnosis.

Beside using cercariae or serum as samples for the diagnosis of schistosomiasis by MALDI-TOF MS, two other studies employed different materials for identification of *Fasciola* spp., the causative agents of fascioliasis [38,39]. One study focused on the analysis of individual components of *Fasciola* spp., while the other one used adult *Fasciola* worms. Mohan et al. [39] conducted a study on the biomedical properties of haemoglobin from *Fasciola gigantica* using MALDI-TOF MS. Extracted peptides were applied to the MALDI target plate and analyzed. The resulting spectra, to which the authors referred as “peptide map” or “peptide mass fingerprints” (PMF), were then queried to a protein databases (e.g., Swiss-Prot) for identification. The authors found that 28.4% of the peptide sequences corresponded to *Fasciola hepatica* haemoglobin. In the second study Sy et al. [38] demonstrated that adult stages of *F. hepatica* and *F. gigantica* can be identified and differentiated with MALDI-TOF MS. For this purpose, the authors subjected the posterior part of the adult worm to molecular analysis and, after protein extraction, to MALDI-TOF MS analysis. Raw spectra of seven *Fasciola gigantica* and one *Fasciola hepatica* samples were analysed and processed to generate species-specific MSPs for both species, which were subsequently integrated into an in-house database for helminth identification. To validate this database, authors performed a blind test using spectra from new samples and obtained a correct identification of 98.7% (74/75) for *F. gigantica* and 100% (3/3) for *F. hepatica* with LSVs above 1.7.

The following table summarizes the different studies published on MALDI-TOF MS for helminths since 2019, as well as the different helminth species and the type of material used (Table 1).

## 4. Effect of Storage Media and Duration

In the field, in research, or in diagnostic laboratories, the lack of common or standardized procedures for sample preservation prior to analysis implies that helminth samples are usually kept under different conditions (e.g., freezing, room temperature, etc.), and storage media (e.g., sodium chloride, ethanol, formalin, RNAlater, etc.) for a short or prolonged period of time before further sample processing [40,41,42,43]. For the purpose of using MALDI-TOF MS as a potential tool for the diagnostic of helminths, it is necessary to use suitable preservative media to avoid the degradation of proteins in order to generate high-quality spectra [20]. Thus far, we identified only two studies that have investigated the influence of different preservative media on MALDI-TOF MS analysis. Wendel and colleagues studied the influence of four storage media (70% ethanol, LC-MS grade water, formalin and 0.45% sodium chloride) on MALDI identification of *T. saginata* proglottids. They reported that all tested media except for formalin were suitable and equally effective for a prolonged storage period of up to 24 weeks. This study was limited by the fact that the comparatively analyzed *T. saginata* proglottids had previously been preserved in sodium chloride for 12 months [22]. Likewise, Mayer-Scholl and colleagues [44] compared two conservation methods (freezing of the native sample vs. storage in ethanol) and found that neither LSVs and nor protein peak patterns were affected after a period of storage of up to six months. However, slight differences in the intensities of the peaks were noticed. In this study, the samples were kept at −20 °C, but the authors did not specifically mention how long the samples were stored in alcohol. Further investigations using different preservation media and different conditions on different helminth species would be helpful for a better understanding and management of samples for MALDI-TOF MS analysis.

## 5. Challenges and Future Research Needs

For reference spectra to be included in databases for helminth identification, it is an essential prerequisite to be sure about the species identification, which is ideally achieved by molecular diagnostic methods in conjunction with microscopic or morphological observation. While MALDI-TOF MS holds promise to be employed in helminths [45,46], it has also been applied for identification of protozoa and different ectoparasites such as ticks [47], lice [48], fleas [49] or bed bugs [50]. As described above, MALDI-TOF-based differentiation seems to be challenging for closely related species, and it might thus be worthwhile to further investigate the use of machine learning (ML) algorithms to further enhance the identification process [36]. This might be particularly useful when analyzing biospecimens (e.g., serum) where pathogen and host proteins occur simultaneously.

Most helminth infections in humans are diagnosed by either urine or stool sample examinations, and the detectable stage are frequently the helminth eggs and sometimes the larvae (e.g., in the case of *Strongyloides stercoralis*), but very rarely parts of the adult worm (e.g., proglottids of *Taenia* spp.). Hence, in the perspective of future applications of MALDI-TOF MS for human helminth diagnosis, upcoming investigations should focus on the analysis of eggs and egg-containing samples (e.g., stool). However, MALDI-TOF MS analysis of stool samples would require a preliminary step of egg purification in order to generate egg-specific spectra that can be used as references. In addition, future studies should also consider the analysis of samples from different geographical locations. Finally, it would be valuable to develop a uniform protocol for sample preservation and for protein extraction, as well as the creation of a universal database for a wide range of parasites. Spectra reproducibility, especially inter-laboratory reproducibility could be solved by standardizing the procedures, from sample storage (by using suitable storage media and conditions allowing non-degradation of proteins), to sample preparation methods (by using a simple protocol). Drug susceptibility testing necessarily involves preliminary identification of the infectious agent in order to determine the appropriate drug for subsequent administration. Therefore, rapid and accurate identification by MALDI-TOF MS would allow for more effective treatment. However, measuring drug sensitivity or monitoring treatment efficacy on adult worms, larvae or eggs would require additional approaches such as real-time cell assay (RTCA) to evaluate changes in worm motility and/or egg hatching [51,52].

## 6. Conclusions

The narrative review highlights the recent advances in helminth diagnosis using MALDI-TOF MS as well as their current limitations. A series of research needs has been identified, which currently hinders the use of this technique as detection method in clinical practice. However, based on the promising available data, it seems likely that further progress will be made in the next years, and that MALDI-TOF MS might also find a potential niche for diagnostics of helminths in the medical and/or veterinary sector.

## Figures and Tables

**Table 1:** Published studies pertaining to the application of MALDI-TOF MS on helminth samples between January 2019 to September 2022.

Pathogens	Analyzed Material					Correct Identification Rate in % [LSV]	References
	Serum of Infected Individuals		Specific Proteins	Larval Stage	Adult Helminth		
**Cestodes**							
***Taenia crassiceps***		√				NA	Diaz-Zaragoza et al., 2020
*Taenia saginata*					√	97.2–99.7% [≥2.5]	Wendel et al., 2021
**Trematodes**							
*Alaria alata*				√		94.73% [≥2]	Kästner et al., 2021
*Cotylurus* sp.				√		65.78% [≥1.7]	Huguenin et al., 2019
*Diplostomum pseudospathaceum*				√			
*Echinostoma revolutum*				√			
*Echinoparyphium* sp.				√			
***Fasciola* spp.:**						98.7–100% [1.73–2.23]	Sy et al., 2020
*F. gigantica*					√		
*F. hepatica*					√		
*Parastrigea* sp.				√		65.78% [≥1.7]	Huguenin et al., 2019
*Petasiger phalacrocoracis*				√			
*Plagiorchis* sp.				√			
*Parastrigea* sp.				√			
*Posthodiplostomum* sp.				√			
*Petasiger phalacrocoracis*				√			
***Schistosoma*: **						42.7% [≥1.7] ≥97% [NA]	Huguenin et al., 2022
*S. bovis*				√			
*S. haematobium*				√			
*S. mansoni*				√			
*S. rodhaini*				√			
*S. bovis* × *S. haematobium (hybrid)*				√			
*Schistosoma japonicum*	√					92–100% [NA]	Huang et al., 2019
*Trichobilharrzia anseri*				√		65.78% [≥1.7]	Huguenin et al., 2019
*Tylodelphys* sp.				√			
**Nematodes**							
*Anisakis* spp.				√		NA	Marzano et al., 2020
*Brugia malayi*		√				NA	Petralia et al., 2022
***Cyatosthomins*: **						89% [1.13–2.44]	Bredtmann et al., 2019
*Cylicostephanus longibursatus*					√		
*Cylicostephanus minutus*					√		
***Dirofilaria* spp.:**						NA 70–100% [NA]	Khanmohammadi et al., 2019 Martini et al., 2019 Nagorny et al., 2019
*D. immitis*	√	√			√		
*D. immitis*		√					
*D. repens*					√		
*Haemonchus contortus*		√			√	NA	Wang et al., 2021
***Trichinella* spp.:**						83.3–100% 100% [≥2]	Karadjian et al., 2020
*T. britovi*				√			
*T. nativa*				√			
*T. patagoniensis*				√			
*T. pseudospiralis*				√			
*T. spiralis*				√			
*Trichinella* sp. *T8*				√			
*Trichinella* sp. *T9*				√			
***Trichuris* spp.:**						100% [1.84–2.36]	Rivero et al., 2022
*Trichuris* sp.					√		
*T. ovis*					√		
*T. suis*					√		
*T. trichiura*					√		
*T. vulpis*					√		

NA: not applicable; LSV: log score value.

## Data Availability

Not applicable.
